# Supplementary material for: Performance and Limitations of Out‐Of‐Distribution Detection for Insect DNA Barcoding
Source: Ecol Evol. 2026 Feb 17;16(2):e73112. doi: 10.1002/ece3.73112 (PMC12912926; doi:10.1002/ece3.73112)
Supplement: Supplementary file 1 — Data S1: ece373112‐sup‐0001‐Supinfo01.docx. [file ECE3-16-e73112-s001.docx]

**Supplementary figures**

Supplementary figure S1. A diagram showing the detailed architecture of the CNN model


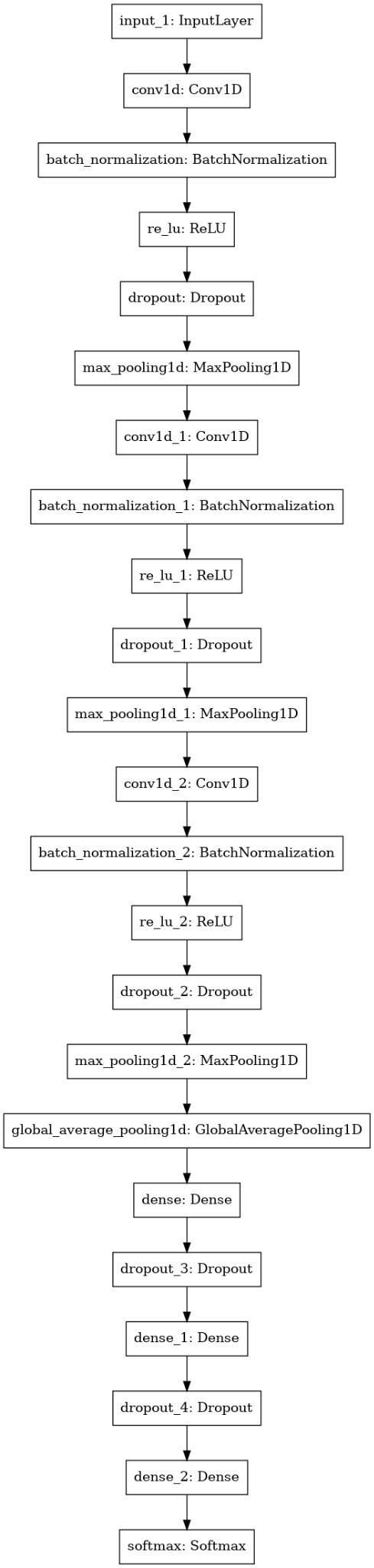


Supplementary figure S2. Exemplary distribution of *g(x)*, showing the outputs of the penultimate FC layer. PCA was applied to reduce the dimensionality for visualization. a) Dots in colors represent in-distribution (ID) samples of different species, while crosses in corresponding colors are class centers; μ_k_. b) the same plots with OOD samples are shown in black dots. ID samples were frequently clustered in linearly separable groups in the intermediate output space, while OOD samples were placed between such groups. Hence, distances from class centers to samples can be used to measure the OOD status of samples.


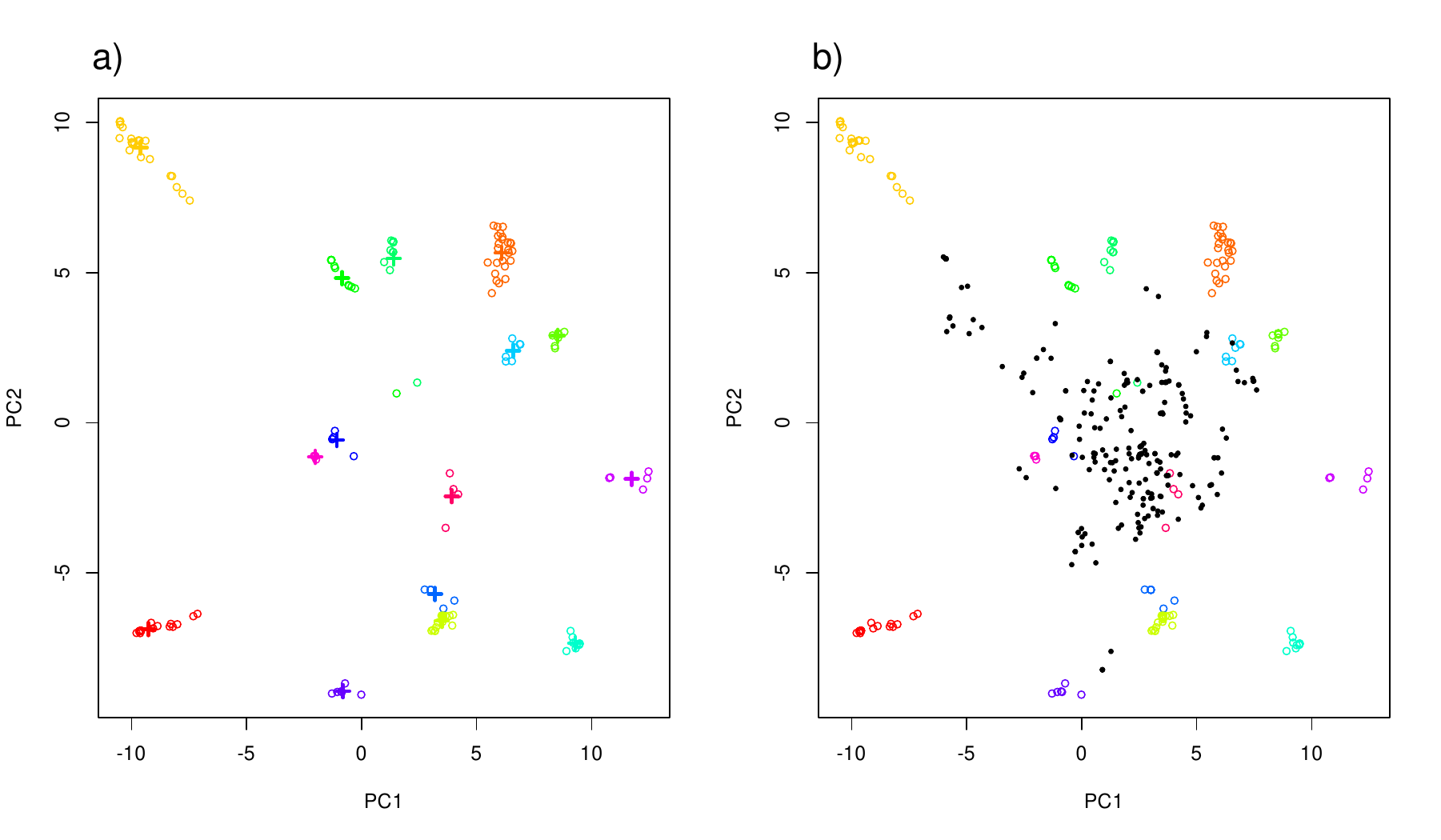


Supplementary figure S3. Distribution of negative energy scores for ID and OOD samples from the Drosophila dataset. Taxon IDs of the OOD samples were assigned based on the predictions of the CNN classifier. Open squares indicate the 95% quantiles of the energy scores of ID samples. Samples with lower negative energy scores with these thresholds were detected as OODs. OOD samples missed by these procedures, such as those with high energy scores in Taxon ID 4, were considered false negatives.


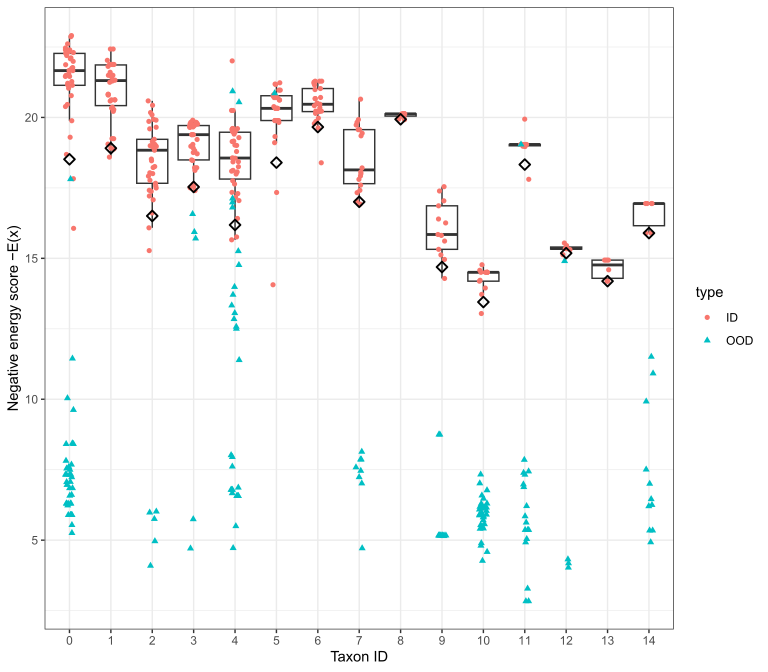


Supplementary figure S4. False negative rates (FNR@95%) of four OOD detection methods and their relationships with fragment lengths. Results on the noiseless sufficient-size dataset are shown. MSP: Maximum Softmax Probability, MV: Majority Voting.


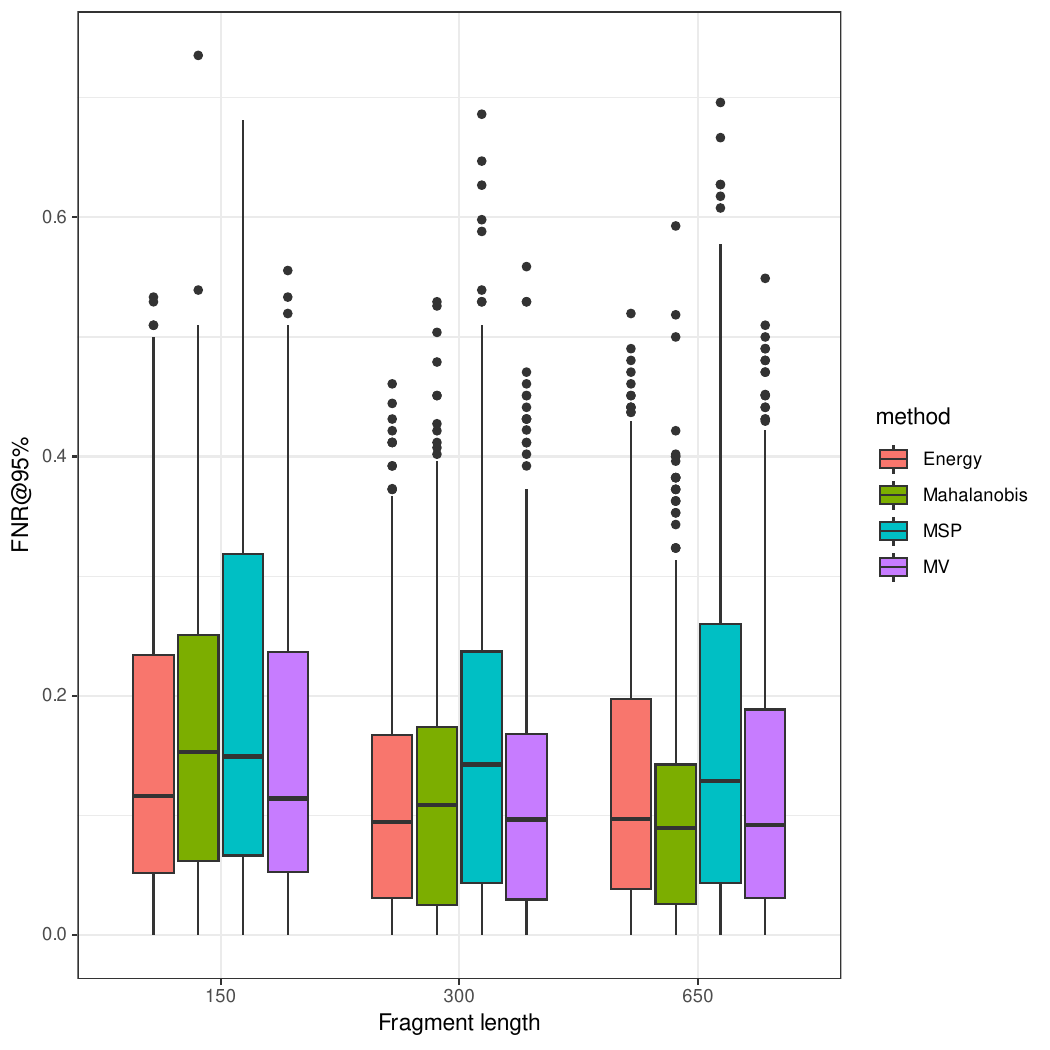


Supplementary table 1. Dataset summary.

| Genus | Dataset Code | Order | Common Name | No.ID.samples | No.ID-species | No.OOD samples |
| --- | --- | --- | --- | --- | --- | --- |
| *Drosophila* | dro15 | Diptera | fruit fly | 1080 | 15 | 162 |
| *Megaselia* | meg42 | Diptera | scuttle fly | 2296 | 42 | 348 |
| *Aedes* | aed19 | Diptera | tiger mosquito | 1169 | 19 | 102 |
| *Atheta* | ath18 | Coleoptera | rove beetle | 707 | 18 | 228 |
| *Cryptocephalus* | cry22 | Coleoptera | leaf beetle | 828 | 22 | 304 |
| *Pterostichus* | pte45 | Coleoptera | ground beetle | 2383 | 44 | 169 |
| *Dolerus* | dol24 | Hymenoptera | sawfly | 907 | 24 | 135 |
| *Megachile* | mch15 | Hymenoptera | leafcutter bee | 649 | 15 | 240 |
| *Lassioglossum* | las68 | Hymenoptera | sweat bee | 2960 | 68 | 910 |
| *Euxoa* | eux40 | Lepidoptera | owlet moth | 1623 | 40 | 778 |
| *Phyllonorycter* | phy53 | Lepidoptera | leaf mining moth | 2086 | 53 | 439 |
| *Acleris* | acl32 | Lepidoptera | leaf roller moth | 1645 | 32 | 138 |
| *Culicoides* | cul26 | Diptera | biting midge | 992 | 26 | 248 |
| *Amara* | ama25 | Coleoptera | sun beetle | 1142 | 25 | 260 |
| *Catocala* | cat62 | Lepidoptera | underwing moth | 2135 | 62 | 355 |
| *Andrena* | and28 | Hymenoptera | mining bee | 873 | 28 | 565 |
| *Bombus* | bom24 | Hymenoptera | bumble bee | 1187 | 24 | 167 |
| *Corynoptera* | cor19 | Diptera | fungus gnat | 1927 | 19 | 23 |
| *Bembidion* | bem39 | Coleoptera | ground beetle | 1053 | 39 | 226 |
| *Caloptilia* | cal17 | Lepidoptera | leaf mining moth | 780 | 17 | 189 |

Supplementary table 2

False negative rates at the 95% threshold for the four OOD detection methods of the deep learning model. The best performing methods are indicated in boldface. The results for a noiseless, sufficiently sized dataset are shown. MSP: Maximum Softmax Probability, MV: Majority Voting.

|  | Database | Sufficient |  |  |  |
| --- | --- | --- | --- | --- | --- |
|  | Method | MSP | Energy | Mahalanobis | MV |
| Noise level | Fragment length |  |  |  |  |
| 0.0 | 650 | 0.169 | 0.13 | **0.11** | 0.128 |
|  | 300 | 0.171 | **0.124** | **0.124** | 0.126 |
|  | 150 | 0.202 | 0.157 | 0.167 | **0.156** |

**Supplementary Methods**

-*Deep learning OOD detection methods*

OOD detection is the task of separating samples into two categories: *IN-DISTRIBUTION,* hereafter, *ID*, which includes samples from classes present in the training data, and *OUT-OF-DISTRIBUTION* or *OOD*, which includes samples from classes NOT present in the training data (Zhang et al. 2024). We employed three methods based on the prediction uncertainty scores: maximum softmax probability (MSP, Hendrycks and Gimpel 2016), energy score (Liu et al. 2020) and Mahalanobis distance score (Lee et al. 2018)

Three OOD scores used in this study were calculated from the output obtained from intermediate fully connected (FC) layers. When the output of the penultimate FC layers was *g(x)*, the following transformation to *g(x)* was applied in the final FC layer:

$$f\left( x \right)=mg\left( x \right)+a$$

Here, *f(x)* is the output of the final FC layer, which is a vector of length equal to the number of classes; *m* is a weight matrix; and *a* is an offset vector. Each of these parameters were optimized in the training process. These intermediate outputs, *f(x)* and *g(x)*, contain useful information for discriminating OODs from ID samples (Supplementary figure S2).

--Maximum softmax probability

The maximum softmax probability (MSP) is commonly used as the prediction probability for neural network classification. The MSP score is defined as a function of the processes of exponentiation and scaling of *f(x)*, the output of the final FC layer, and its maximum value:

$$MSP\left( x \right)=\max_{k} \left( \frac{exp(f_{k}(x))}{\sum_{k=1}^{K} exp(f_{k}\left( x \right))} \right)$$

Here, $f_{k}\left( x \right)$ is the k-th component of the vector *f(x)*. The kth class that yields the MSP (i.e. $\underset{k}{\mathrm{argmax}}$) is a predicted assignment of sample *x*. Importantly, this predicted class is chosen only from the classes present in the training dataset, regardless of whether the sample is of the OOD type. Hence, OOD detection is required to avoid the erroneous assignment of an OOD sample to a known class. Hendrycks and Gimpel (2016) proposed MSP as a metric for prediction uncertainty and showed that MSP scores of OOD samples were consistently lower than those of the ID sample, and a cutoff by a threshold of prediction probability helped to successfully detect OOD samples.

--Energy score

Liu et al. (2020) introduced the "energy score" of a neural network model for OOD detection. The (negative) log energy score of a neural network is defined as

$$NegativeEnergyScore\left( x \right)=-E\left( x \right)=log\left( \sum_{k=1}^{K} \exp\left( f_{k}\left( x \right) \right) \right)$$

The negative log energy score is the logarithm of the softmax denominator in *MSP(x)*. *E(x)* is interpreted as “free energy” of the data point, *x*, and has lower values for ID samples. Therefore, the *negative* energy score, -E(x), of OOD samples was consistently lower than that of ID samples (Figure S3) The energy score is also interpreted as the relative log-likelihood score of a model given a sample *x*, *Pr(x|model)*. Low scores of OOD samples indicate that the likelihood of obtaining such samples is less for models trained only with ID samples. Liu et al. (2020) reported that the threshold of sample energy values outperformed the softmax probability for multiple OOD detection tasks.

--Mahalanobis distance

Lee et al. (2018) developed a distance-based OOD detection method. The Mahalanobis distance of a sample from a class center is defined as

$$d_{k}\left( x \right)={(g\left( x \right)-\hat{\mu_{k}})}^{T}\hat{\Sigma}^{-1}(g\left( x \right)-\hat{\mu_{k}})$$

where ${}_{k}$ is the k-th class center value, and Σ is a variance-covariance matrix of *g(x)*. Mahalanobis distance measures the distance from the k-th class center, assuming that the distribution of *g(x)* follows a multivariate normal distribution with a mean μ_k_ and a single variance-covariance matrix, $\hat{\Sigma}^{-1}$ , which are empirically estimated from a distribution of *g(x)* in a training data set. Lee et al. (2018) proposed the following negative Mahalanobis distance to the closest distribution center as an uncertainty metric for OOD detection:

$$M\left( x \right)=\max_{k} (-d_{k}\left( x \right))$$

--Majority voting for OOD detection

In addition to independent OOD detection procedures with the above metrics, we devised a process for OOD detection with majority voting for the above three detectors. With this approach, a sample was treated as an OOD sample if two of the three methods "vote" for the presence of OOD.

*One-dimensional GradCAM and grad-energy map for DNA barcoding*

GradCAM localizes the region of importance by measuring the effects of CNN features on the classification probabilities. Specifically, the GradCAM score on window *w,* is defined as

$$L_{w, GradCAM}=\mathrm{ReLU}\left( \sum_{n=1}^{N} \alpha_{n}A_{n,w} \right)$$

$L_{w, GradCAM}$ is a weighted average of *N* CNN features, $A_{n,w}$, calculated on the window *w* with the weight$\alpha_{n}$. The weight is a feature importance, measured as an averaged partial derivative of MSP(x) with respect to$A_{n,w}$, $\alpha_{n}=\frac{1}{W}\sum_{w=1}^{W} \frac{\partial MSP(x)}{\partial A_{n,w}}$, where *W* is the total number of windows on a sequence. An interpretation of importance is that when a unit change in a CNN feature ($A_{n,w}$) results in a significant change in the prediction probability (MSP(x)), $A_{n,w}$ is considered to be important in the prediction process. We also implemented an activation map of the energy score to visualize the region responsible for OOD detection decisions by replacing the gradient of the MSP(x) in the weight calculation with the gradient of the energy score.

$$L_{w, Grad-Energy}=\mathrm{ReLU}\left( \sum_{n=1}^{N} \beta_{n}A_{n,w} \right)$$

Here, the weight $\beta_{n}$ is defined as $\beta_{n}=\frac{1}{W}\sum_{w=1}^{W} \frac{\partial E(x)}{\partial A_{n,w}}$. In this case, the effect of the CNN features on the energy score was measured. In the current study, the window size was set to 8 bp, resulting in 85 windows in a 680 bp fragment.
